# Developments and Prospects in Imperative Underexploited Vegetable Legumes Breeding: A Review

**DOI:** 10.3390/ijms21249615

**Published:** 2020-12-02

**Authors:** Sandeep Kaur Dhaliwal, Akshay Talukdar, Ashish Gautam, Pankaj Sharma, Vinay Sharma, Prashant Kaushik

**Affiliations:** 1 Department of Plant Breeding and Genetics, Punjab Agricultural University, Ludhiana 141004, India; sandeep2-pbg@pau.edu (S.K.D.); pankaj-pbg@pau.edu (P.S.); 2 Division of Genetics, Indian Agricultural Research Institute, New Delhi 110012, India; akshay.talukdar@icar.gov.in; 3 Department of Genetics and Plant Breeding, G.B. Pant University of Agriculture and Technology, Pantnagar, Uttarakhand 263145, India; gautam.ashish801@gmail.com; 4 International Crops Research Institute for the Semi-Arid Tropics (ICRISAT), Hyderabad 502324, India; s.vinay@cgiar.org; 5 Nagano University, Ueda 386-0031, Japan

**Keywords:** underexploited legumes, vegetable breeding, pre-breeding, molecular markers, QTLs

## Abstract

Vegetable legumes are an essential source of carbohydrates, vitamins, and minerals, along with health-promoting bioactive chemicals. The demand for the use of either fresh or processed vegetable legumes is continually expanding on account of the growing consumer awareness about their well-balanced diet. Therefore, sustaining optimum yields of vegetable legumes is extremely important. Here we seek to present d etails of prospects of underexploited vegetable legumes for food availability, accessibility, and improved livelihood utilization. So far research attention was mainly focused on pulse legumes’ performance as compared to vegetable legumes. Wild and cultivated vegetable legumes vary morphologically across diverse habitats. This could make them less known, underutilized, and underexploited, and make them a promising potential nutritional source in developing nations where malnutrition still exists. Research efforts are required to promote underexploited vegetable legumes, for improving their use to feed the ever-increasing population in the future. In view of all the above points, here we have discussed underexploited vegetable legumes with tremendous potential; namely, vegetable pigeon pea (*Cajanus cajan*), cluster bean (*Cyamopsis tetragonoloba*), winged bean (*Psophocarpus tetragonolobus*), dolichos bean (*Lablab purpureus)*, and cowpea (*Vigna unguiculata*), thereby covering the progress related to various aspects such as pre-breeding, molecular markers, quantitative trait locus (QTLs), genomics, and genetic engineering. Overall, this review has summarized the information related to advancements in the breeding of vegetable legumes which will ultimately help in ensuring food and nutritional security in developing nations.

## 1. Introduction

Agriculture has been under growing pressure to produce sufficient food, feed, and biofuel on scarce land for the planet’s predicted nine billion people by 2050 [1]. Growing adequate food for the ever-increasing population in the climate change scenario is a major challenge for food security. In this case, underexploited vegetable legumes make an erudite argument for contribution to rural people’s dietary needs. These days, consideration of underexploited vegetable legumes is growing new protein sources to meet the ever-increasing demand for vegetable proteins [2]. The term underexploited/underutilized crop refers to the group of cultivated and wild species that have limited global market potential and are sometimes deemed as under-used [3]. Globally, underexploited legumes are known as nutritious resources and can be intended to improve health [4] and minimize disease risks [5]. They have a specific profile with high nutrient and protein content alternatives to maintain farmers’ livelihoods and soil protection [6]. In comparison, underexploited legumes are a less utilized potential source to improve protein and micronutrient content in comparison to cereals which provided ample calories but insufficient micronutrients [7]. Legumes are mainly cultivated as pulse (seeds) or animal fodder. However, some members of the legume family are cultivated for their pods and immature seeds to cook as a vegetable [8].

Vegetable legumes have unique organoleptic qualities and are usually regarded as important sources of carbohydrates, minerals, vitamins, and health-promoting bioactive compounds. In contrast, legumes are also known to carry some antinutritional factors like lectins, phytic acid, saponins, and vicine [9]. Moreover, legumes are characterized by their ability to develop in a symbiotic relationship with nitrogen-fixing bacteria and therefore are also used as soil-enriching green manure [10].

Pigeon pea (*Cajanus cajan*) cultivation is mostly confined to South Asia and to East Africa, which is one of the most malnourished regions of the globe. Whereas, the cluster bean or guar is an incredibly drought-tolerant annual legume crop grown because of its use as a vegetable, green manure, and forage [11]. On the other hand, winged bean is important in that nearly all components of the plant can be consumed, from the seeds, pods, and flowers, to the foliage as well as the tuberous roots, with the stems as well as leaves used as fodder [12]. Therefore, in countries where protein deficiency is high, or access to meat protein is low, winged bean is a candidate for helping to diversify diets and significantly improve nutrition.

Furthermore, legumes, when consumed as vegetables, contain more water compared to pulses. Therefore, the soluble carbohydrates are higher and starch content is lower in the vegetable legumes, making them much more palatable compared to dry pulses [13]. Additionally, vegetable legumes are more abundant sources of health-promoting compounds like carotenoids, vitamin A, chlorophyll, phenolics, and vitamin C. For that reason, their consumption is generally supposed to make for far healthier nutrition [14]. Besides, vegetable legumes are short-season crops with a short shelf life [15]. On the other hand, the usage of processed vegetable legume products is continuously growing due to the increasing awareness of their well-balanced nutrition and high health-promoting compounds content [16].

Not many efforts have incorporated legumes for their beneficial use as vegetable legumes. So, there is an utmost need to improve vegetable legumes with the help of modern breeding technologies. There are many underexploited species of vegetable legumes which are available as local accessions and landraces and are being consumed as vegetables [17]. So, these accessions are the valuable genetic variability basket from which we can extract the traits of interest for the improvement of vegetable legumes. Traits like high yield, early podding, year-round availability, long and large green pods, better shelf life, and biotic and abiotic stress resistance are among the most desired characteristics in the vegetable legume breeding programs [18]. Econometric research awareness and policymakers’ attentions on underexploited legumes will be needed for further diversification of nutritional profiles and enhancements of human nutrition. There are currently about 150 cultivated crops, and only 30 edible species are often used for global diets, the majority of which are cereal-based, and which in developing countries rely especially on rainfed agriculture [19]. Almost all of these crops cannot withstand abiotic stresses due to global climate change [20]. However, underexploited legumes have tremendous potential to withstand harsh conditions that cannot be ignored, which will help in mitigating nutritional insecurity. Over the past decade, it has been seen that cultivated legumes having a narrow genetic base and continuous use of a few elite breeding lines are the key causes affecting genetic improvement in breeding programs. To fulfill the needs of plant-based micronutrients and rejuvenation of soil health, breeding programs need to adopt a new approach. Crop wild relatives (CWRs) have become an ideal source of novel alleles for a range of important traits needed for improvement in breeding programs. In this regard, recently, in a pigeon pea breeding program utilizing wild *Cajanus platycarpus* sp., a stable promising trait-specific introgression line (IL) CPL 87119 has been identified. It showed higher potential for yield and nutrient-rich traits with a broad genetic base [21]. Efforts are required in this direction to increase the quality of other underexploited legumes.

In recent years, advances in next-generation sequencing (NGS) methods and a steep decrease in sequencing cost offers an incredible opportunity for the improvement of vegetable legumes [22]. Moreover, the number of publications that deal with the breeding aspects of underexploited vegetable legumes are diminutive in comparison to papers dealing with grain legumes [23]. To best of our knowledge, a review paper centered on highlighting the breeding development and thereby, outlining the crucial breakthroughs in underexploited vegetable legumes is lacking in the international scientific literature. Further, combining the available knowledge on this subject will help in developing an in-depth understanding of the breeding of vegetable legumes. Recognizing this gap, this review paper provides a detailed overview of all elements connected to the underexploited vegetable legumes.

## 2. Crop Wild Relatives

Comprehending the relationship of crop plants and their wild relatives is a tremendous focus of plant breeders. This expertise is of excellent worth in dissecting the process of crop domestication by determining and employing wild relatives for crop development. Breeding programs, as well as germplasm characterization research, over the years have discovered that the cultivated plants, generally, have a relatively reduced tolerance to stresses compared to their wild relatives [24]. The one-dimensional prospect for enhanced yield has been hypothesized to guide metabolic supply allocation in the direction of accelerated progress, thereby overlooking other traits. On the other hand, breeding bottlenecks have relatively reduced inherited deviation of contemporary vegetation as well as led to the loss of genes created by crop wild relatives (CWRs). Although genes are identified for disease and insect pest resistance, they have been seen as negatively correlated with yield [25]. However, it has been discovered that breeding for disease and insect pest resistance traits might be attained without having the demand for crop yield compromised. CWRs present several arrays of attributes with the chance to minimize the amount of yield loss as a direct result of biotic and abiotic stresses. The traits present in the CWRs can be introduced into cultivated varieties using conventional breeding approaches (if there is sexual compatibility), transgenesis, and more. In this direction, introgression of characteristics of interest originating out of a CWR to a cultivated type via consistent breeding would encounter linkage drag [26]. Below we have discussed the breeding objectives and use of CWRs for the six underexploited vegetable legumes addressed in this review in detail.

### 2.1. Vegetable Pigeon Pea (Cajanus cajan)

Vegetable pigeon pea possesses favorable agronomic qualities compared to other main grain legumes, and its wild relatives show promise in providing vital adaptive traits [27]. Higher investment in phenotypic and genotypic characterization and evaluation for the adaptive traits present in the CWR, symbolize equally immediate steps for the improvement of vegetable pigeon pea. Additional unrepresented species like *Cajanus crassus* and *Cajanus scarabaeoides*, are essential for developing germplasm collections for the improvement of important traits of the cultivated pigeon pea [28]. As strategies for the effective utilization of extensive diversity of plant genetic resources, conservation, collection, and accessibility of even more distant relatives of vegetable pigeon pea will be rewarding. In this direction, *C. scarabaeoides*, as well as *C. platycarpus*, are recognized as demonstrating potential related to the adaptation to climatic change [29].

### 2.2. Cluster Bean (Cyamopsis Tetragonoloba)

The genus *Cyamopsis* has four important members, *C. tetragonoloba*, *Cyamopsis serrata*, *Cyamopsis senegalensis*, and *Cyamopsis dentata* [30]. It has been accepted that the cultivated *C. tetragonoloba*, was developed from *C. senegalensis*, which is a drought tolerant African species. Breeding programs are mainly focused on breeding for high nutrition and dietary fiber, but also for improving the gum content (galactomannans) in the endosperm (90%). Wild relatives *C. serrata*, *C. senegalensis*, and *C. tetragonoloba* are diploid with chromosome number 2n = 2x = 14 [31]. Recently genome size was determined for the three *Cyamopsis* species with the help of flow cytometry. It was observed that the genome size of wild species, *C. serrata*, was approximately double (979.6 Mbp) that of cultivated cluster bean *C. tetragonoloba* (580.9 Mbp) whereas *C. senegalensis* (943.4 Mbp) had genome size intermediate between these two species. This information is critical to further implement specific tools for crossing wild relatives, which are the storehouse of many useful genes [32].

### 2.3. Winged Bean (Psophocarpus tetragonolobus)

The untamed progenitor of winged bean has remained somewhat enigmatic due to the absence of wild *Psophocarpus* in Asia, leading to one suggestion that the true wild progenitor is now extinct [33]. Morphological phylogenetic analyses of the nine species in the genus have come to mixed conclusions. Probably the closest wild species to winged bean scanned by the most recent morphological analysis places winged bean alongside *Psophocarpus scandens* and *Psophocarpus palustris*. Few efforts to cross winged bean with various members of the genus have been reported, however, one profitable cross between winged bean as well as *P. scandens* has been manufactured following many attempts [34]. Yet, molecular phylogenetic analysis is lacking. Identifying the true progenitor(s) may assist in the breeding of winged bean and could be necessary to understand the genetic changes associated with domestication. Relatively few studies have investigated the domestication genetics of legumes, except for scientific studies of the winged bean [35]. Therefore, little is known about the genes as well as alleles that were under selection by early farmers. This may contribute to the observation that genetic enhancement of legumes remains slow between distant relative and other crops.

### 2.4. Dolichos Bean (Lablab purpureus)

The dolichos bean is unquestionably of African origin; the only taxon known is subspecies *uncinatus*, which is widespread in tropical Africa. In Africa, *Lablab’s* wild ancestor grows in hilly areas and coastal lowlands in southern, eastern, and western Africa. Its beans are too small and are not eaten. The cultivated form is known in Egypt, Sudan, and both East and West Africa [36]. Hence, it is essential to evaluate the breeding potential of parents and to select good combiners in the *Lablab* bean. Good results of any breeding program rely on an assortment of parents [37]. Nature is depicted by this study and the magnitude of gene action regarding evolving connected characteristics of parents and their offspring. Gene actions may show heterosis in F_1_ or linkage in other generations [38]. Certainly, *Lablab’s* undomesticated ancestor is still scattered across, and endemic to, much of tropical Africa [39]. Additionally, the wild forms collected from India and analyzed through molecular marker research were discovered genetically positioned intermediately between wild and cultivated forms. Still, there is a lot of consideration, which thinks the origin centers are Africa and Asia [40].

### 2.5. Cowpea (Vigna Unguiculata)

Cowpea is indigenous to West Africa and weedy and wild types exist in many parts of the region. It typically suffers from considerable losses caused by diseases and pests [41]. Though its tolerance to drought and heat is superior to other crops, predictions for climate change in the region suggest that there is a need for germplasm with even higher levels of adaptation to these abiotic and biotic stresses [42]. To be able to enhance tolerance to these stresses, this project seeks to tap the genetic diversity present in the wild relatives of cowpea. Cowpea wild relatives are the storehouse of important genes for disease and insect resistance, and the genes of abiotic stress resistance [43]. Moreover, CWRs of cowpea are important for maintaining genetic diversity and protecting against loss of germplasms because of genetic vulnerability. Several reports have shown that the weedy subspecies of cowpea (*V. unguiculata* subsp. *dekindtiana*, *stenophylla*, etc.) are easy to hybridize with the popular cultivated varieties. F_1_ hybrids are also known to use a degree of vigor over the parent genotypes. The effective crossing between cultivated cowpea (*V. unguiculata*) varieties and their wild distant relatives varies based on genotypes and species [44]. Members of the var. *pubescens* have been known to confer some degree of insect resistance on cowpea owing to the presence of hairs (hence the title *pubescens*) on the plants. Transferring the hairiness trait from the wild lines to the cultivated varieties is essential for the development of insect resistance and consequently, avoidance of pathogens transmitted by such insects [45]. In the past, many studies were focused on determining the cross-compatibility between cultivated cowpea and wild relatives, finding out the reproductive potential, and also heterosis of the F_1_ hybrids from these crosses [46].

## 3. Pre-Breeding

Pre-breeding activities in mobilizing novel alleles for cultivar development from wild relatives have been routinely involved in breeding programs [47]. Pre-breeding includes all activities directed at the identification of attractive crop traits including genes, as well as the consequent transfer of theirs into an excellent set of parents for extra choice [48]. The procedure identifies helpful characteristic(s) or maybe genes that could be exploited in cultivar growth. For vegetable legumes improvement, enough genetic diversity is present in wild relatives and landraces, which carry several helpful genes for cultivar improvement [49]. Pre-breeding activities must be initiated to produce new genetic variability using wild relatives and promising landraces for usage by the breeders in crop advancement programs. Pre-breeding must concentrate on the constant source of significant variability into the breeding pipeline to build new high yielding cultivars with a broad genetic base [50]. Though pre-breeding is helpful to enrich the primary gene pool for cultivar improvement, it is a time-consuming and challenging affair also. In this direction, linkage drag associated with wild relatives can make the pre-breeding tasks far more troublesome. Genomic-assisted pre-breeding is going to help to conquer the linkage drag and can facilitate the focused transfer of valuable genes/segments from wild relatives [51]. Therefore, it is important to understand all the above-mentioned factors for pre-breeding to exploit the available genetic variability for developing improved cultivars with a broad genetic base in vegetable legumes.

## 4. Molecular Markers from Diversity to QTLs

The directed evolution towards the improvement of existing germplasm requires tracking the desired traits to bring them together. Earlier, the phenotype served as a tracker of traits which is now replaced by more reliable DNA markers. The amalgam of breeding and molecular biology has made deeper insight into traits possible, as the genome is dug with more markers, one gets closer to a gene controlling trait. The complete bouquet of genes, quantitative trait loci (QTLs), and molecular markers linked to traits are put together for reliable marker-assisted breeding. The advancement in marker technology is slower in legumes, particularly for vegetable type than cereals, earning them the title of orphan crops. In the evolutionary line of molecular markers, the first generation markers, namely, restriction fragment length polymorphism (RFLP), random amplification of polymorphic DNA (RAPD), and amplified fragment length polymorphism (AFLP), generating information of many loci in one go, have been employed mainly in diversity analysis of pigeon pea [52], cluster bean [53], winged bean [54], dolichos bean [55], and cowpea [56,57]. On the other hand, sequence-based markers, namely, simple sequence repeats (SSRs), single nucleotide polymorphism (SNPs), and their modifications, being more reliable and reproducible, are employed in linkage mapping, trait mapping, and fine-mapping studies. SSR marker systems are available in pigeon pea [58], cluster bean [59], winged bean [33], and cowpea [60], whereas inter-species SSRs have been used in dolichos bean [61,62]. SNPs are markers of choice owing to their ubiquitous nature and abundance in the genome. SNPs have been identified in pigeon pea [63], cluster bean [64], winged bean [65], dolichos bean [66], and cowpea [67] for potent use in genomics assisted breeding. Once the choice of marker system is established, it draws a path for mapping traits of interest leading towards fine mapping and cloning. A selected set of mapped QTLs in pigeon pea and cowpea being comparatively rich in molecular resources along with important marker systems in cluster bean, winged bean, and dolichos bean, which are growing towards mapping studies are compiled (Table 1).

The pigeon pea crop has a rich set of molecular markers and QTLs for its improvement. Dense molecular linkage maps have been developed using SSR markers [68] and SNP markers [69]. A consensus genetic map was developed using previously published maps and four maps generated from four mapping populations. The consensus map comprised 339 SSRs spanning over a genetic distance of 1059 cM [68]. The consensus map is available, but traits of vegetable pigeon pea such as pod color, pod size, pod weight, and tenderness remained undiscovered on a genomic scale. The SSR and SNP markers can be used for reducing generations for obtaining desired recombinants through marker-assisted backcross breeding and de novo mapping studies. The QTLs mapped in pulse pigeon pea can be transferred to vegetable types such as sterility mosaic resistance, determinacy, earliness, Fusarium wilt resistance, and fertility restoration are compiled in Table 1. Fusarium wilt is a deadly disease limiting crop yield in Eastern and Southern Africa, whereas sterility mosaic virus is another devastating disease transmitted by a mite (*Aceria cajani*). The use of genomic resistance is a cost-effective alternative against chemical control for fungal diseases and viral vectors. Three QTLs, namely, *qFW11.1*, *qFW11.2*, and *qFW11.3* for Fusarium wilt resistance [70] and a candidate gene *CcLG11* for sterility mosaic virus were mapped which can serve as potent resistance donors [71].

The development of an efficient marker system is slower in cluster bean, ultimately reducing growth in revealing genetic control of loci across the genome. The orphan crop gained the focus of breeders mainly due to gum produced by it which is useful commercially in textile and other allied industries. Thus, its use as a vegetable crop remained shadowed. However, the advancements in marker technology over the years could be to utilized in the improvement of the crop for vegetable purpose. In the HES 1401 cultivar a total of 16,476 expressed *sequence* tags (EST) were reported, and it is the first step in the omics era for the crop [72]. Subsequently, these EST sequences were explored for the existence of SSRs with MISA (microsatellite identification tool) software (Thomas Thiel @ the Plant Genome Resources Center) which resulted in obtaining 187 SSRs [73]. Later on, the same set of EST was utilized to develop SSRs and was used to validate 32 diverse genotypes [74]. Recently, a total of 73,934 sequences were developed in the GG-4 variety by Mi seq NGS technology [59]. Later in the process, these sequences were mined for SSRs and resulting in finding 15,399 SSRs [59]. In future, marker development studies in vegetable legumes would need to be explored to generate genomic resources and genic markers as has been done in other crops.

Winged bean is a neglected crop in terms of genomic resources and molecular breeding. Molecular characterization was attempted for 24 accessions of winged bean using 13 RAPD and 7 ISSR markers [75]. It was found that ISSR markers were more promising in comparison with RAPD markers. It was an era of development of omic resources in other crops when this study was conducted for cluster bean, this clearly indicates its neglect in molecular breeding. The further studies also used ISSR markers for diversity analysis [55], until the breakthrough study in which transcriptome sequencing was performed on the ”Ibadan Local-1” cultivar and 1900 SSRs markers were discovered [76]. More transcriptomes were sequenced after this study by Vatanparast et al. [65] and Wong et al. [77] giving rise to 12,956 and 9682 SSRs, respectively. Besides, a total of 5190 SNPs was also generated in the study [73]. Among these, a total of 20 microsatellite primer pairs were validated on 53 accessions of cluster bean for their use in molecular studies [33]. There is still plenty of scope for development of saturated linkage maps of SSR and SNP markers for the improvement of cluster bean.

Dolichos bean has a variety of uses such as vegetable, medicinal plant, and fodder, but its molecular resources remain unrevealed. The first genetic linkage was made harboring 127 RFLP and 91 RAPD using the second filial generation of cross “Rongai (cultivated) and CPI 24973 (wild species)’’. A total of 17 linkage groups spanning 1610 cM were generated in the study [78]. Due to scarcity of microsatellite markers in a crop, SSRs from related crops were tested for transferability for use in molecular breeding. Transferability was tested for 50 SSRs from soybean [79], genic SSRs from cowpea [80], 42 SSRs from soybean, *Medicago truncatula*, and chickpea [61], and 134 SSRs from French bean, mung bean, cowpea, faba bean, and moth bean [62]. The transferable SSRs from related crops were used for mapping photoperiod insensitivity and determinate growth habit in dolichos bean using bulk segregant analysis [81]. Bulks and population derived from the cross of two phenotypic extremes, namely, GNIB21 and GP189, result in identification of a *PvTFLy1*, a locus controlling the determinate habit of growth linked with photoperiod sensitivity. These traits are found linked in common bean and soybean as well, and thought to be controlled by mutation of *Dt1* and *E3* homologs, in dolichos bean. Although transferable markers were reliable, there was the urgency of generating markers from its genome to cover the entire genome. Therefore, a total of 459 ESTs of dolichos bean have been obtained from the National Center for Biotechnology Information (NCBI) and searched for the presence of microsatellites. Thus, 22 SSRs were discovered in a total of 420 unigenes and validated on a set of 24 accessions of dolichos bean [82]. Association mapping is a strategy of using historic linkages to associate markers with traits. It is particularly useful in dolichos bean owing to its self-pollinated nature, as a result of which fewer recombination and widely spaced markers could be used. A set of 234 SSRs (mostly in-house designed) were used in association with mapping for identifying genetic control of days to 50% flowering, fresh pod number per plant, and fresh pod weight per plant. Three markers, namely, KTD 200, KTD 130, and KTD 273 were found to be associated with traits, respectively explaining more than 10 percent of phenotypic variation in each case [83].

Cowpea is a much-explored crop at the molecular level in comparison to the crops discussed above. Dense molecular maps and QTLs are available for its genetic improvement. The first genetic linkage map of vegetable cowpea (asparagus bean) comprising 191 SNP and 184 SSR loci distributed on 11 linkage groups was developed covering 745 cM of total length [84]. The development of such genetic maps is a foundation stone for future breeding work. Pod tenderness and sweet taste are essential traits of vegetable cowpea, QTL mapping of these traits using backcross and F_2_ generation of the cross of JP81610 and JP89083 was performed. Pod tenderness was found to be under genetic control of three QTLs explaining up to 50 percent phenotypic variation whereas two QTLs were obtained for total soluble solids (TSS) [85]. The QTLs for tender pods on LG 7 co-localized with pod length QTLs, which played an important role in the domestication of cowpea. In another study, major horticulturally important traits were mapped using recombinant inbred line RILs and it was found that QTLs for days to flowering, nodes to the first flower, leaf senescence were clustered together on LG11 whereas QTLs for pod number per plants were scattered on various linkage groups [86]. Pod length and biotic and abiotic stresses faced by cowpea were mapped using genome-wide association studies (GWAS) which take into account historical linkages to map the traits that are compiled in Table 1. These mapping efforts not only detected the genomic regions and candidate genes but also enabled the development and validation of trait linked markers and facilitate their use in future breeding programs.

## 5. Genomic and Transcriptomic Resources

Breeding objectives for crops, including vegetable legumes, are constant over the years but approaches towards achieving the goals are ever-changing. The availability of genomic and transcriptomic resources has changed the ways of shaping genomes and creating innovative possibilities to alter the genome for the desired phenotype (Table 2). The era of genomics was revolutionary for legumes by sequencing of model legume species such as *Glycine max* [108] which served as the legume genome reference. The dissection of sequenced genomes of model plants aids the understanding of evolution, important gene families, and re-arrangements in the structure of chromosomes in related crops [109]. Pigeon pea genome [110], dolichos bean [111], and cowpea [112] have been sequenced providing insights into agriculturally essential genes.

The sequenced genomes of legumes and the model species would accelerate genomic advancements through comparative genomics in cluster bean, dolichos bean, and winged bean, which are still at a rudimentary stage. Furthermore, genome sequencing was one of the important milestones in comparative genetics making it easier for scientists to compare the genomes, transfer of traits and markers, identifying orthologous and paralogous genes, and more in-depth insight into evolution and domestication [105]. Legumes stand as shuffled, deleted, and doubled genomes from one common ancestor with a common monophyletic family making transferability of genomic information possible [113]. Comparative genome analysis of asparagus bean with soybean, adzuki bean, and mung bean using SLAF (specific length amplified fragment sequencing) markers revealed conserved genomic regions, and offer support in assembling genome sequence [105]. Salinity tolerance mechanisms of *Strophostyles helvola*, a wild inhabitant of beaches in North America was revealed by transcriptome sequencing [114].

More and more genomes get sequenced owing to the reduction in the cost of sequencing and new reference genomes become available, stimulating resequencing projects [115]. Resequencing of wild and cultivated germplasm in various crops has been initiated to get closer to genes underlying essential traits. One of the cost-effective resequencing or de novo sequencing strategy is the reduced representation library approach [116]. One of the widely used dimensions of this technology is genotyping by sequencing (GBS); GBS has been used for mapping the traits of interest by deep sequencing of parents and multiplexed sequencing of large mapping populations in one go. Fusarium wilt resistance and fertility restoration was mapped in pigeon pea [70,117], and potyvirus resistance mapping [118] and mapping aphid resistance in cowpea, using GBS [119]. The technology has enormous potential in mapping and genomic studies of cluster bean, winged bean, and dolichos bean owing to lack of genomic tools, as the platform is flexible. The sequencing of the population under investigation is carried out, which exempts it from ascertainment bias [120].

The primary outcome of genomic and transcriptomic studies is a large set of SNP markers that can be used for high throughput genotyping assays. SNPs fit best for most high throughput genotyping because they are omnipresent in eukaryotic genomes, cost-effective, automated platforms, and allele calling and data analysis are simple owing to their bi-allelic nature [121]. In pigeon pea, the 56k Axiom SNP chip was used for mapping seed quality and high-selfing flower traits [122]. The genotyping assay has also been used for mapping pod length in cowpea [103] and understanding molecular mechanisms governing incompatible and compatible reaction against *Striga* in cowpea [123]. In parallel, the latest pigeon pea SNP chip ”CcSNPnks” has enormous potential for use in mapping studies as the SNPs originate from unique genes, conserved genes of pigeon pea with related crops and other agriculturally important genes [124]. These developed chips can also be employed in the winged bean, cluster bean, dolichos bean, and other orphan legumes for testing their suitability for molecular studies. Adopting such genomics and transcriptomic methods could overcome several limitations of traditional breeding and improve the precision and efficiency of crop breeding procedures.

## 6. Transgenics and Genome Editing

Plant breeding offers extensive opportunities for the creation of desirable variation through hybridization and mutation. The scale of hybridization is limited, and the transfer of genomic information is impossible across reproductively incompatible genotypes by conventional techniques. Genetic engineering serves the purpose of transferring alien genes across species which otherwise are not feasible through conventional breeding. The routinely employed transformation technique in legumes is *Agrobacterium*-mediated gene transfer [142] owing to their dicot nature [143]. The major bottleneck in legume transgenic is the regeneration of explants due to their recalcitrant nature [144]. Among various explants, the use of young embryonic axes [145], cotyledonary nodes [146], and immature tissues and preconditioning of seedling with thidiazuron [147] have proved successful recovery. A few selected examples of economically important traits integrated into legume genome include, rice chitinase pigeon pea [146], *dhdps-r1* (increased lysine) pigeon pea [148], *P5CSF129A* (salt-tolerant) pigeon pea [149], *cry1Ac* pigeon pea [150], *cry1AcF* pigeon pea [151], c*ry1AcF* dolichos bean [152], *aAI-1* (insect resistant) cowpea [153], *cry1Ab* cowpea [154], and soybean isoflavone synthase gene in cowpea [155]. On the other hand, there are no reports of the transformation of cluster bean and winged bean for economically important traits in the context of vegetable type characteristics.

Although, the regeneration and transformation protocols in both crops have been standardized [35]. In winged bean, successful organogenesis has been obtained from callus derived from cotyledons [156], epicotyls [157], excised segments of leaf [158], and protoplasts [159]. Further, in cluster bean, cotyledonary nodes, cotyledons [160], hypocotyls, and epicotyls have reported successful regeneration [161]. In view of available protocols for transformation and improvement, cluster bean and winged bean can be exploited for genetic engineering for useful, economical traits such as biotic and abiotic resistance genes, quality traits, yield-enhancing genes, and growth habit controlling genes. However, some anti-nutrient traits from these legumes have been extracted, such as cowpea trypsin inhibitor against rice stem borers in transformed rice plants [162]. The winged bean lysine-rich protein (WBLRP) isolated from winged bean was patented [163] and hexaploid wheat was transformed by WBLRP comprising expression vector which increased lysine content by 2-3-fold in transgenic wheat [164].

**Figure 1:**
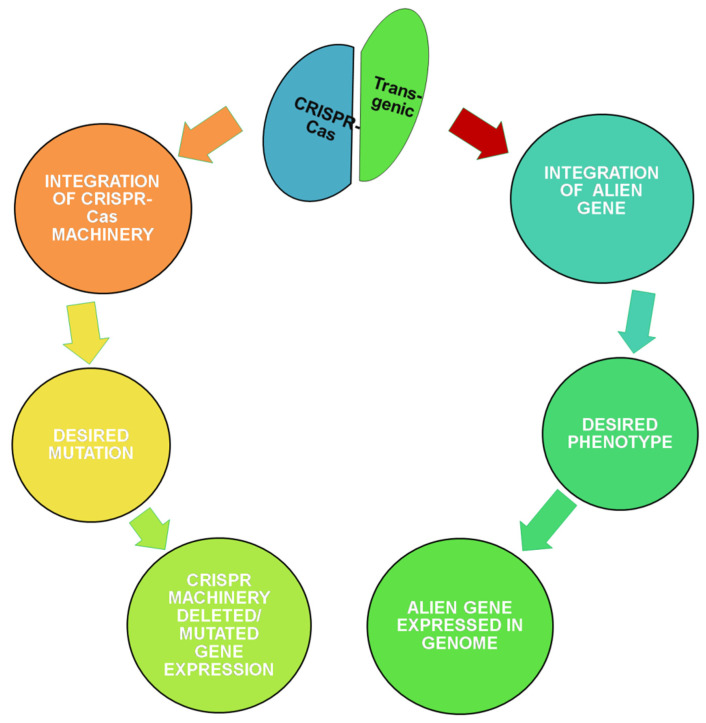
Illustration of CRISPR-Cas versus transgenic methodology and outcomes.

One of the promising techniques of post-transcriptional gene silencing is RNA interference (RNAi) in which ds-RNA molecules prevent gene expression, conferring resistance to pathogenic nucleic acids and regulating the expression of protein translating mRNAs [165]. This technology has emerged as a promising technique in plants to fight against invading pathogenic viruses. The host plant is engineered to express ds-RNA, which inhibits expression of the complementary gene in pathogens [166]. RNAi is a potent technology for insect resistance in legumes through silencing genes essential for insect survival. In tobacco, RNAi mediated gene silencing was achieved against *Helicoverpa armigera* through vector construct carrying *HaAce1* gene (*H. armigera* acetylcholinesterase) in the backbone of *HaAce1*-preamiRNA1 from *Arabidopsis* controlled by CaMV 35S promoter against *H.a armigera* [167]. With the availability of cloned sequences of insect and pathogen genes, a similar approach can be employed in vegetable legumes for insect and pathogen resistance [168]. The transgenic crops face biosafety issues that vary from country to country and struggle through the journey from lab to land [169]. Genome editing is emerging as a widely adopted targeted approach and does not fall under the category of genetically engineered crops in the USA [170]. However, some countries still lack clarity between the two technologies (Figure 1).

Genome editing can be accomplished by site-specific double-strand breaks in DNA caused by homing endonucleases (HEs) [171], zinc finger nucleases (ZFNs) [172], transcription activator-like effector nucleases (TALENs) [173], and clustered regularly interspaced short palindromic repeat and CRISPR-associated protein (CRISPR-Cas type II) [174]. The *homology-directed repair* (HDR) is a template mediated repair technique, in which exogenous template sequences can be supplied to introduce desired sequence change. Mostly plant viruses serve the purpose of delivery of template sequence as in the case of potato, a geminivirus replicon (GVR) is employed to deliver sequence-specific nucleases (SSNs) targeting acetolactate synthase 1 (ALS1) gene and customized repair templates constructed to induce point mutation for herbicide resistance within the locus [175]. Among the various technologies for inducing double-strand breaks, CRISPR-Cas has proven to be more site-specific with the least off-targets, easier to use, and thus widely adopted for genome editing [176]. The CRISPR-Cas system is ready to be exploited in legumes for desirable mutations in the gene of interest with optimization of the protocol in model species *Arabidopsis* and *Nicotiana benthamiana*. The pea early browning virus was used as a delivery system of Cas 9 and guide RNA in model species, and this virus is known to cause disease in 30 species along with the members of family Leguminosae [177,178]. Therefore, the same virus can be engineered for legume vegetables to express desirable guide RNA sequence homologous to the site to be mutated. In future, using genome editing methods will lead to the development of non-genetically modified crops, with desired traits.

## 7. Future Prospects

Most developed legumes used for food are consumed as grain seeds known as pulses. Nevertheless, the growth of some legume species is aimed at the consumption of theirs as vegetables [179]. From a health perspective, legumes are considered valuable sources of plant protein, carbohydrates, essential minerals, vitamins, and phytochemicals. Importantly, legume vegetables comprise a low-fat diet with high proportions of digestible proteins. Vegetable legumes provide several raw materials for products ranging from coatings for cloth and paper to eco-friendly plastics as well as biofuel. Legumes draw the interest of scientists seeking to exploit their nutritional resources [180]. Legumes have extreme diversity and various stress tolerance capabilities; it might enhance food and food security in low-income areas of Africa [20]. Exploring underexploited legumes’ nutritional value can overcome malnutrition, especially in Sub-Saharan Africa, in developing nations [181]. Similarly, nutrient strength can be analyzed and used in formulations for the food-to-food approach [182]. Study especially on wild relatives of underexploited legumes for qualitative and quantitative traits and their domestication will contribute significantly to nutrition security. In this respect, underexploited legumes of reasonable nutritional value can be harnessed with other local food products for recipe development based on nutritional requirements [181].

**Figure 2:**
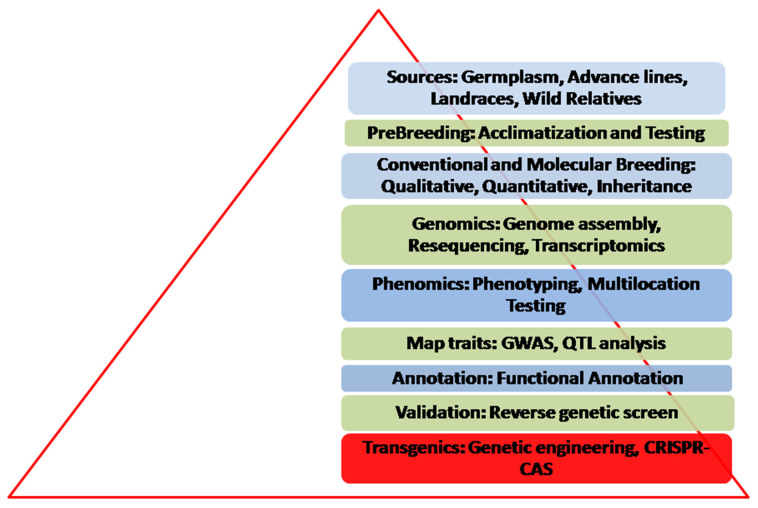
Stages and best approaches for consideration in vegetable legumes improvement.

Average yields of legumes have changed significantly in recent decades. This achievement was due in part, to the breeding of better performing hybrids developed by combining CWRs inbred plant genomes. Moreover, in recent decades breakthroughs in genomic technologies and availability of vegetable legumes’ draft genome sequence knowledge accelerates the breeding vision. However, to support the world’s increasing population, it will be essential to sustain the rate of increase in vegetable legumes production. This challenge will probably be addressed through better farming, much more reliable seed supplies, plus more stable markets as well by the application of genomics. Most commercially important plant phenotypes depend on the interactions of large numbers of genes. With the advent of genomics tools, breeders can characterize the allelic characteristics of their particular germplasm in detail that is exquisite throughout the breeding program and therefore retaining the many useful allele combinations. Overall, the process and its various steps can be summarized as in Figure 2.

Integration of genomics tools with conventional breeding methods would assist breeders in their attempts to design and properly choose the best combinations of chromosome segments, alleles, and genes offered in the related species to fulfill the requirements for crop production enhancement.

## Figures and Tables

**Table 1:** Molecular markers and mapped quantitative trait loci (QTLs) in vegetable legumes.

Crop	Molecular Marker/QTL	Source	Trait/Objective	Reference
Pigeon pea	*qSMD4* major QTL and minor QTLs	F_2_ (ICP 8863 × ICPL 20097 TTB 7 × ICP 7035)	Sterility mosaic resistance	[87]
	13 QTLs for six traits	(Pusa Dwarf × HDM04-1)	Earliness, plant type, high-density linkage map	[88]
	339 SSR, 4 QTLs	F_2_ (ICPB 2049 × ICPL 99050, ICPA 2043 × ICPR 3467, ICPA 2039 × ICPR 2447, ICPA 2043 × ICPR 2671)	Linkage map, fertility restoration	[68]
	*CcTFL1* gene	F_2_ (ICPL 85010 × ICP 15774)	Determinacy	[89]
	*C.cajan*_01839 for sterility mosaic, *C. cajan*_03203 for Fusarium wilt	RILs (ICPL 20096 × ICPL 332)	Fusarium wilt, sterility mosaic disease	[90]
	421 hypervariable SSRs from a genome sequence	94 genotypes	Diversity Analysis, Hybrid Purity Testing, Trait Mapping	[91]
	3 major QTLs (CcLG11)	F_2_, RIL (ICPL 20096 × ICPL 332, ICPL 20097 × ICP 8863, ICP 8863 × ICPL 87119)	Sterility mosaic resistance	[71]
	*Dt1* locus, Indel marker from*CcTFL1* gene	F_2_ (ICP 5529 × ICP 11605)	Determinacy	[92]
	547 SNP (bead-array), 319 SNP (RAD), 65 SSR	F_2_ (Asha × UPAS, Pusa Dwarf × H2001-4, Pusa Dwarf × HDM04-1)	Molecular linkage map	[69]
	CcLG08 carry major QTL	F_2_ (ICPA 2039 × ICPL 87119	Fertility restoration	[92]
	CcLG07 (8 QTLs), SNP S7_14185076 (linked to 4 traits)	BC (ICPL 87119 × ICPW 15613, ICPL 87119 × ICPW 29)	Yield related traits	[93]
Cluster bean	16,476 EST	HES 1401	cDNA library from seeds	[72]
	*L19*, *D1*, *AB7* and *QLTY 3* (Bacterial blight) and *OPQ 20*,*OPD10*, *OPD14*,*OPQ 12*,*OPAC 8* and *OPF 9* (drought tolerance) QTLs	HG 75 × PNB (Bacterial blight) and HG 563 × PNB (drought tolerance)	Mapping Bacterial blight resistance and drought tolerance	[94]
	5 RAPD	35 genotypes	RAPD and ISSR cloning and sequencing	[95]
	100 SSRs	32 genotypes	Validation of SSRs	[74]
	15,399 SSRs	GG-4 variety	Sequencing by Miseq NGS	[59]
Winged bean	13 RAPD and 7 ISSR	24 accessions	Molecular characterization	[75]
	100 ISSR	45 accessions	Diversity analysis	[54]
	1900 SSRs	Ibadan Local-1	Transcriptome sequencing -Illumina HiSeq 2500	[76]
	12,956 SSRs, 5190 SNPs	2 accessions	Transcriptome sequencing- Roche 454 Genome Sequencer FLX	[65]
	9682 SSR	6 accessions	Transcriptome sequencing-Illumina MiSeq	[77]
	20 SSRs	53 accessions	Primer design from in house assembled transcriptome using primer3	[33]
Dolichos bean	127 RFLP, 91 RAPD	Rongai (cultivar) × CPI 24973 (wild) -17 linkage groups, 1610 cM	F_2_ population for genetic linkage map	[78]
	41 main effect QTLs (22 for growth phenological traits and 19 for fruit traits)	Meidou2012 × ‘Nanhui23	Growth phenological and fruit traits	[96]
	40 QTLs (8.1 to 55.0% variation)	(Meidou2012 × Nanhui 23).	Inflorescence length traits	[97]
	21 SSR	13 genotypes	Transferability of SSRs from French bean/diversity analysis	[98]
	60 SNPs, 16 InDels.	Sequencing polymorphic genic segments of 9 parents	Allele-specific PCR primers	[99]
	22 SSRs	420 unigenes	479 EST from NCBI for SSR mining	[82]
	42 SSRs		Transferability of markers from soybean, *Medicago truncatula*, green gram chickpea	[61]
	134 SSRs	143 genotypes	Transferability of SSRs from French bean, mung bean, cowpea, faba bean, and moth bean/diversity analysis	[62]
	9 QTLs using 234 SSRs	64 accessions for GWAS	Fresh pod yield mapping	[83]
	*PvTFLy1* locus	GNIB21 × GP189	Photoperiod responsive flowering	[81]
Cowpea/Asparagus bean	191 SNP and 184 SSR loci	RILs (ZN016 × Zhijiang282)	Molecular linkage map	[84]
	3 QTLs for pod tenderness, 2 QTLs for total soluble solid	F_2_ and BC (P81610 × JP89083)	Pod tenderness and total soluble solid	[85]
	Major QTLs on LG 11	RILs (ZN016 × ZJ282)	Days to first flowering (FLD), leaf senescence (LS), nodes to first flower (NFF), and pod number per plant (PN)	[86]
	39 SNPs using GWAS	95 accessions of asparagus bean	Drought tolerance	[100]
	18 SNPs using GWAS	95 asparagus bean, 4 African cowpea accessions	Fusarium wilt	[101]
	QTLS on LG 1,4,7	F_2_ and BC (JP81610 × TVnu-457)	Pod fiber content and pod shattering	[102]
	72 SNPs using GWAS	RILs (ZN016 × Zhijiang282)	Pod length	[103]
	17,996 SNPs using RAD sequencing, QTLS on LG4, 5, 6, 7, 9, 10, 11	F_2:3_ (Green pod cowpea × Xiabao II)	High-density SNP map and yield traits	[104]
	5225 SNP markers by SLAR-seq	F_2_ (Dubai bean × Ningjiang 3)	High-density map by sequencing	[105]
	*Ruv2* locus	F_2_ and RILs (ZN016 × Zhijiang282)	Rust resistance	[106]
	*qCel7.1*, *qHem7.1*, and *qLig7.1*	F_2_ (JP81610 × TVnu-457)	Pod fiber content	[107]

**Table 2:** Transcriptomic and valuable genomic resources in legumes (pigeon pea, cluster bean, winged bean, dolichos bean, and cowpea).

Crop	Objective	Description	Genetic Improvement of Vegetable Type	Platform	Reference
Pigeon pea	Transcriptome seq	50,566 SSRs, 12,000 SNPs, 0.12 million unique sequences and 150.8 million sequence reads	Enhancing genomic resources	Roche FLX/454	[125]
	RNA-seq	1.696 million reads, 3771 SSRs	To target protein-coding and regulatory genes	Roche 454 GS-FLX	[126]
	Gene expression atlas (CcGEA)	590.84 million paired-end data from RNA-Seq, 28 793 genes, regulatory genes, i.e., pollen-specific (*SF3*), sucrose–proton symporter	To target protein-coding and regulatory genes	Illumina HiSeq 2000	[127]
	Comparative transcriptome	*Cajanus cajan* (L.) and *Cajanus platycarpus* (Benth.) sequence revealed 0.11 million transcripts, 82% annotated	Valuable data from wild sources	Illumina Hi-Seq 2500	[128]
	WRKY characterization	94 WRKY genes characterized and validated phylogenetically three groups (I, II, III)	Elucidating stress-responsive machinery	qRT-PCR	[129]
	Axiom SNP array	56K SNPs from 104 genotypes	SNP genotyping	Axiom Affymetrix	[130]
	CcSNPnkssnp chip for Affymetrix GeneTitan	62k SNPs from conserved, unique, and stress resistance genes	SNP typing	Illumina Hiseq	[124]
Cluster bean	seedling (Ibadan Local-1)	1900 SSRs and 1800 conserved orthologous loci	Stimulating genomics accelerated breeding in winged bean	Illumina HiSeq 2500	[76]
	RNA-Seq	5773 SSR, 3594 SNPs, 62,146 unigenes with mean 679 bp length, and 11,000 genes annotated for biochemical pathways	To target protein-coding and regulatory genes	Illumina HiSeq 2500	[131]
	RNA-Seq	127,706 transcripts, 48,007 non-redundant unigenes, 79% annotations,8687 SSRs	To target protein coding and regulatory genes	Illumina paired end sequencing	[132]
	CbLncRNAdb database	lncRNAs, miRNAs identification, and characterization	Understanding the stress mechanism	http://cabgrid.res.in/cblncrnadb.	[133]
	Whole-genome sequencing	1859 SSRs from 1091 scaffolds constituting 60% genome of the cluster bean	Towards complete genome assembly	Illumina and Oxford nanopore	[134]
	Whole-genome assembly	1.2 Gb genomic reads comprising 50% genome of cluster bean (Illumina and Oxford nanopore)	Towards complete genome assembly	Illumina HiSeq 2500	[135]
	Genome sequencing of GG-4	15,399 SSRs generated	Towards complete genome assembly	Illumina MiSeq	[59]
Winged bean	CPP34 (PI 491423) and CPP37 (PI 639033) accessions	16,115 total contigs, 12,956 SSRs and 5190 SNPs developed	To target protein-coding and regulatory genes	Roche 454 Genome Sequencer FLX	[65]
	Tissue specific (leaf, pod root, and reproductive tissues)	198,554 contigs, 24,598 SSR motifs detected	Library of various tissues available for digging important traits	Illumina MiSeq	[77]
	Tannin controlling genes	1235 contigs expressed differentially	Identification of candidates	Illumina Nextseq 500	[136]
Dolichos bean	ORCAE-AOCC	Genomic portal for orphan crops such as dolichos bean	Information for molecular studies		[137]
Cowpea	Chilling tolerance	ICE1-CBF3-COR id cold-responsive cascade present in asparagus bean	Engineering cold-tolerant genotypes	Illumina Hiseq2500	[138]
	Molecular mechanism of chilling injury	Redox reactions enzymes, energy metabolism enzymes, and transcription factors, i.e., *WRKY*, *MYB*, *bHLH*, *NAC*, and *ERF* are involved in chilling injury	To plan genetic improvement by an understanding mechanism of chilling injury	Illumina HiSeq2500	[139]
	Transformable cowpea genotypes	Tissue-specific data special emphasis on reproductive organs	Genetic improvement and mapping studies	Illumina Hiseq 2500	[140]
	SNP chip-Cowpea iSelect Consortium Array	51 128 SNPs obtained by WGS sequencing of 37 different cowpea accessions	High throughput genotyping	Illumina HiSeq 2500	[141]

## Abbreviations

QTLs	Quantitative trait loci
NGS	Next-generation sequencing
CWRs	Crop wild relatives
RFLP	Restriction fragment length polymorphism
RAPD	Random amplification of polymorphic DNA
AFLP	Amplified fragment length polymorphism
SSRs	Simple sequence repeats
SNPs	Single nucleotide polymorphism
EST	Expressed *sequence* tag
*ISSR*	Inter simple sequence repeat
NCBI	National Center for Biotechnology Information
GWAS	Genome-wide association studies
SLAF	Specific length amplified fragment sequencing
GBS	Genotyping-by-sequencing
WBLRP	Winged bean lysine-rich protein
RNAi	RNA interference
HEs	Homing endonucleases
ZFNs	Zinc finger nucleases
CRISPR-Cas	Clustered regularly interspaced short palindromic repeat and CRISPR-associated protein
HDR	*Homology directed repair*
GVR	Geminivirus replicon
