# Correction: Dhaliwal et al. Developments and Prospects in Imperative Underexploited Vegetable Legumes Breeding: A Review. *Int. J. Mol. Sci.* 2020, *21*, 9615

**DOI:** 10.3390/ijms25137052

**Published:** 2024-06-27

**Authors:** Sandeep Kaur Dhaliwal, Akshay Talukdar, Ashish Gautam, Pankaj Sharma, Vinay Sharma, Prashant Kaushik

**Affiliations:** 1 Department of Plant Breeding and Genetics, Punjab Agricultural University, Ludhiana 141004, India; sandeep2-pbg@pau.edu (S.K.D.); pankaj-pbg@pau.edu (P.S.); 2 Division of Genetics, Indian Agricultural Research Institute, New Delhi 110012, India; akshay.talukdar@icar.gov.in; 3 Department of Genetics and Plant Breeding, G.B. Pant University of Agriculture and Technology, Pantnagar, Uttarakhand 263145, India; gautam.ashish801@gmail.com; 4 International Crops Research Institute for the Semi-Arid Tropics (ICRISAT), Hyderabad 502324, India; s.vinay@cgiar.org; 5 Nagano University, Ueda 386-0031, Japan

In the publication [1], there was an error regarding the affiliation of Prashant Kaushik. The affiliation “Instituto de Conservación y Mejora de la Agrodiversidad Valenciana, Universitat Politècnica de València, 46022 Valencia, Spain” has been removed, as per the institution’s request. The authors state that the scientific conclusions are unaffected. This correction was approved by the Academic Editor. The original publication has also been updated.
